# Stability and flexibility in chromatin structure and transcription underlies memory CD8 T-cell differentiation

**DOI:** 10.12688/f1000research.18211.1

**Published:** 2019-07-31

**Authors:** Huitian Diao, Matthew Pipkin

**Affiliations:** 1 Department of Immunology and Microbiology, The Scripps Research Institute, Jupiter, FL, USA

**Keywords:** Memory CD8 T cells, chromatin structure, transcriptional control

## Abstract

The process by which naïve CD8 T cells become activated, accumulate, and terminally differentiate as well as develop into memory cytotoxic T lymphocytes (CTLs) is central to the development of potent and durable immunity to intracellular infections and tumors. In this review, we discuss recent studies that have elucidated ancestries of short-lived and memory CTLs during infection, others that have shed light on gene expression programs manifest in individual responding cells and chromatin remodeling events, remodeling factors, and conventional DNA-binding transcription factors that stabilize the differentiated states after activation of naïve CD8 T cells. Several models have been proposed to conceptualize how naïve cells become memory CD8 T cells. A parsimonious solution is that initial naïve cell activation induces metastable gene expression in nascent CTLs, which act as progenitor cells that stochastically diverge along pathways that are self-reinforcing and result in shorter- versus longer-lived CTL progeny. Deciphering how regulatory factors establish and reinforce these pathways in CD8 T cells could potentially guide their use in immunotherapeutic contexts.

## Introduction

During a prototypical acute intracellular infection that will be cleared, naïve antigen-specific CD8 T cells become activated and their progeny accumulates dramatically, a period generally referred to as the “effector” phase. Near the point of maximal accumulation, cells in the responding population manifest substantial phenotypic and functional heterogeneity. As the infection clears, most effector cells die and the population “contracts”. Cells that survive this period ultimately give rise to an array of memory CD8 T-cell subsets ^1^.

**Figure 1.**
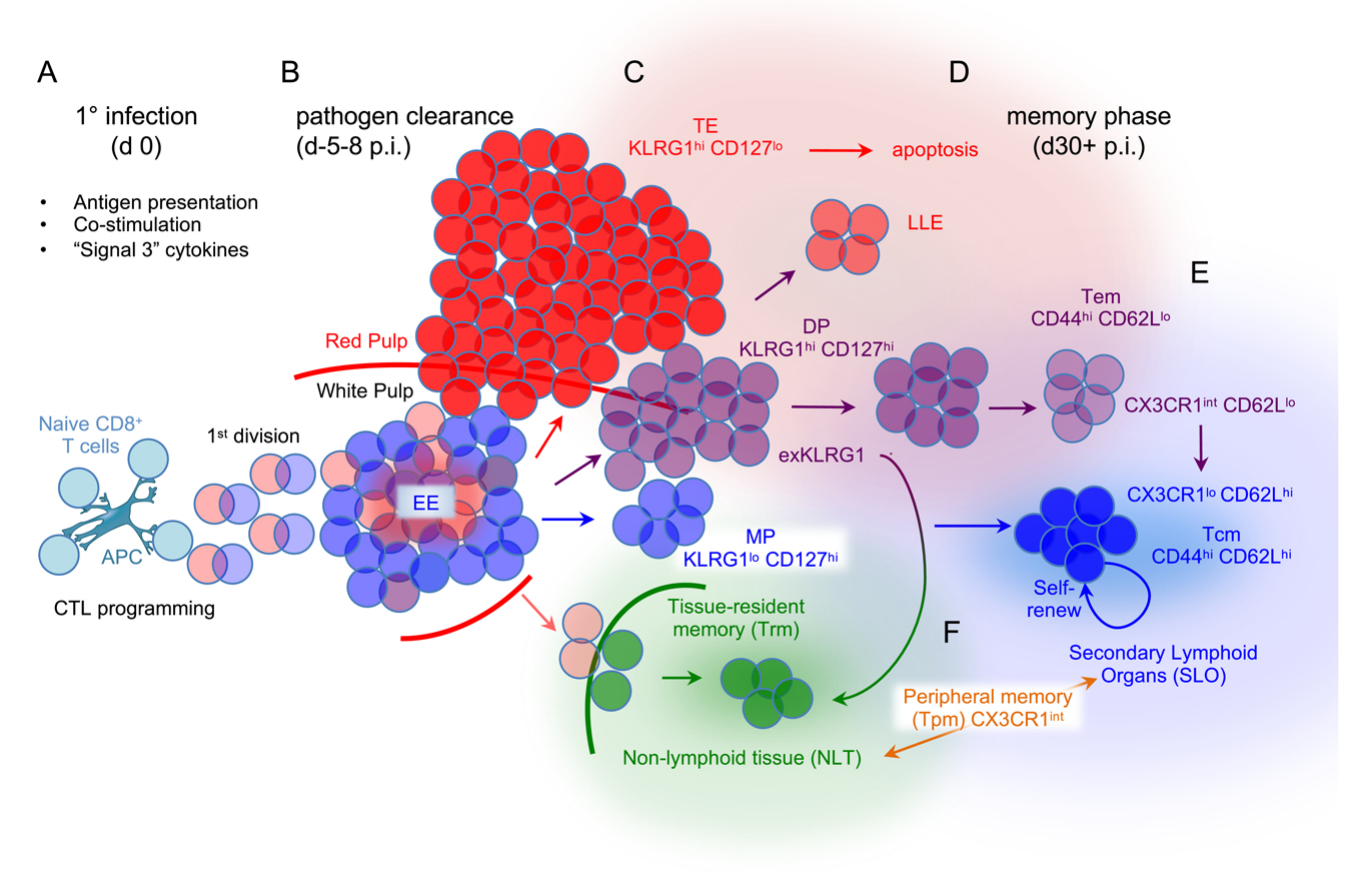
Patterns and inter-relationships of effector and memory CD8 T-cell subsets induced by acute intracellular infection. ( **A**) Antigen presentation, co-stimulation, and additional inflammatory signals induce multiple individual naïve CD8 T cells to undergo a prototypical pattern of geometric expansion. ( **B**) Individual cells within the nascent CTL population of early effector (EE) cells differentiate along any one of multiple trajectories. ( **C**) Multiple phenotypic subsets with distinct memory CD8 T-cell potentials are detectable at the peak response, near the time when most pathogen has been eliminated. Cells that are KLRG1 ^hi^ CD127 ^lo^ have the shortest half-lives after the infection resolves and are referred to as short-lived effector cells (SLECs) or simply terminal effector (TE) CD8 T cells (red). Conversely, KLRG1 ^lo^ CD127 ^hi^ cells are termed memory precursor (MP) effector CD8 T cells (light blue) because they most efficiently generate memory CD8 T cells. However, some double-positive (DP) effector cells that are KLRG1 ^hi^ CD127 ^hi^ ^9^ (purple) downregulate KLRG1 and give rise to memory CD8 T cells. Trm precursors (light green) derived from KLRG1 ^lo^ intermediates in the spleen begin populating non-lymphoid tissues (NLTs) near the peak response. ( **D**) Most TE cells persist poorly into the memory phase. At early memory time points, some KLRG1 ^hi^ cells persist and have been termed effector-like memory cells or long-lived effector (LLE) cells but they wane over time. Tem cells preferentially localize in the vasculature (light red background), some of which convert into Tcm cells (dark blue) that reside in secondary lymphoid organs (light blue background) later during the memory phase. Arrows indicate the general ancestry of the different cell populations and are colored according to the main classes of effector and memory CTL subsets.

Many excellent recent reviews have comprehensively outlined the tapestry and importance of distinct memory CD8 T-cell subsets that arise after infection ^2– 4^. An illustration of the main effector and memory CD8 T-cell subsets in mice depicts their general inter-relationships ( Figure 1) ( Table 1). Memory T cells are classically categorized into central memory T (Tcm) cells, which localize in secondary lymphoid organs (SLOs), and effector memory T (Tem) cells, which recirculate between peripheral tissues and SLOs ^4^. However, at early memory time points, a substantial fraction of the classically defined Tem cells are more effector-like and have been termed effector-like memory cells or long-lived effector (LLE) cells ^5, 6^. Moreover, another subset of classic Tem cells, called peripheral memory T cells, has been delineated as those that recirculate through peripheral tissues via SLOs and has been distinguished from Tem cells that do not recirculate ^7^. In addition, memory T cells that enter and stably reside within tissues have been defined as tissue resident memory (Trm) cells ^8^. Further emphasizing the diversity of memory T-cell subsets is that analysis of human CD8 T cells using cytometry by time of flight has demonstrated that substantial heterogeneity exists between individual cells defined classically as Tcm and Tem cells ^9^. The extent to which all of these phenotypically distinguishable populations of effector and memory T cells comprise stable cell “lineages” is an open set of questions ^3^.

**Table 1.** Key definitions.

**Effector phase:** Time period between the initial infection and when the accumulation of effector cells has peaked. **Contraction phase:** Time period between the peak accumulation of effector cells and when the decreasing effector population numbers have stabilized. **Memory phase:** Time period after pathogen clearance and when the effector cell population has contracted and the antigen-specific cell numbers have stabilized. **Effector cells:** The antigen-activated cells that expand during infection and then die during contraction of the response as pathogen is cleared. **Memory cells:** The stable populations of antigen-specific cells that persist after the effector cell population undergoes contraction. **Early effector (EE) cells:** KLRG1 ^lo^ CD127 ^lo^ cells defined around the time of peak cellular accumulation in response to infection. EE cells retain potential to give rise to terminal effector (TE), double-positive (DP), and memory precursor (MP) cells and ultimately memory T cells. **Terminal effector (TE) or short-lived effector cells:** KLRG1 ^hi^ CD127 ^lo^ cells identified around the time of peak cellular accumulation in response to infection. TE cells are prone to apoptosis during contraction and manifest very weak persistence into the memory phase and weak secondary proliferative capacity upon re-stimulation. **Memory precursor (MP) effector cells:** KLRG1 ^lo^ CD127 ^hi^ cells identified around the time of peak cellular accumulation in response to infection. MP cells efficiently give rise to effector and central memory T cells (Tcm) and manifest strong capacity for persistence and secondary proliferation upon re-stimulation. **Double-positive (DP) effector cells:** KLRG1 ^hi^ CD127 ^hi^ cells defined around the time of peak cellular accumulation in response to infection. Intermediate capacity to contribute to effector memory and Tcm. **Tcm cells:** CD62L ^hi^ CCR7 ^hi^ CD44 ^hi^ (also CD127 ^hi^ and KLRG1 ^lo^ and CD27 ^hi^ and CX3CR1 ^lo^) cells defined after expanded T-cell numbers following infection have contracted and stabilized. Mainly reside in secondary lymphoid organs, exhibit lower constitutive expression of effector molecules, and manifest strongest proliferation upon re-stimulation. **Effector memory T (Tem) cells:** CD62L ^lo^ CCR7 ^lo^ CD44 ^hi^ (also CD127 ^hi^ and KLRG1 ^lo/hi^ and CD27 ^lo^ and CX3CR1 ^hi^) cells defined after expanded T-cell numbers following infection have contracted and stabilized. Mainly reside in vasculature and intravascular spaces, exhibit higher constitutive expression of effector molecules, and manifest less strong proliferation upon re-stimulation compared with Tcm cells. **Peripheral memory T (Tpm) cells:** CX3CR1 ^int^ cells defined after expanded T-cell numbers following infection have contracted and stabilized. Tpm cells are located in both intravascular spaces and recirculating through secondary lymphoid organs and exhibit strong homeostatic renewal. **Tissue resident memory (Trm) cells:** Operationally defined antigen-specific cells that enter non-lymphoid tissues during the effector phase, that are non-vascular-associated, and that do not recirculate. Trm cells have variable phenotypes depending on their host tissues but are frequently CD69 ^+^ and CD103 ^+^. **Long-lived effector (LLE) or effector-like memory (ELM) cells:** LLE cells are KLRG1 ^hi^ CD127 ^hi/lo^ (and CD62L ^lo^) and are mainly CD27 ^lo^ and CD43 ^lo^ (defined as ELM with these markers), probably correspond to most CD27 ^lo^ CX3CR1 ^hi^ cells, and are most frequent at early times of the memory phase. LLE/ELM cells have strong protective capacity and expression of effector molecules but weak capacity for proliferation upon secondary antigen stimulation.

Although a generally agreed upon concept is that memory CD8 T cells derive from effector cells, this general explanation is somewhat unsatisfying because of the semantics in defining what an “effector” cell is ^11– 13^. Phenotypically distinct populations of cells that arise in the effector phase differ in their propensity to form specific types of memory cytotoxic T lymphocytes (CTLs). The phenotypes of cells representing some of these populations are relatively stable and do not readily interconvert whereas others do so more easily ^7, 9, 14, 15^, which likely reflects a spectrum of differentiated states that, on the one end, are terminally differentiated and have relatively short-term roles and, on the other, are stem cell–like and participate in populating and re-populating multiple memory cell niches during iterative infections over time. It is still unclear exactly how all of these differentiated states are initially established and how they are maintained.

Here, we discuss recent studies that have helped to define how activated CD8 T cells terminally differentiate or become memory CD8 T cells, and we focus specifically on the regulation of gene expression and chromatin structure in distinct effector CD8 T-cell populations. Our conclusion is a model that incorporates many of these observations and that might help to clarify how memory CD8 T cells develop from activated cells in the effector phase.

## The descent of memory T cells: individual naïve CD8 T cells initiate memory CD8 T-cell programming rapidly and stochastically undergo terminal differentiation

A brief encounter of T-cell receptors (TCRs) on naïve CD8 T cells with their cognate peptide–major histocompatibility complex together with co-stimulation is sufficient to induce a complete program of memory cell differentiation ^16, 17^. Individual naïve T cells have the potential to differentiate into all phenotypic effector cell subsets and ultimately memory CD8 T cells ^9, 18, 19^. Aspects of this decision could be programmed during the first naïve cell division, as antigen-presenting cell contact establishes molecular asymmetry in nascent daughter T cells which is associated with their ultimate fate ^20, 21^, and cells that have undergone their first cell division exhibit distinct single-cell mRNA expression profiles that can be correlated with either gene expression signatures from mature KLRG1 ^hi^ IL-7Rα (CD127) ^lo^ terminal effector (TE) CD8 T cells at the peak response, or from memory CD8 T cells ^22, 23^. However, the gene expression profiles in single cells 4 days later are neither strongly distinct between each other nor analogous to the profiles observed after the initial cell division. The expression profiles in single cells on day 4 are also distinct from those in mature TE and memory CD8 T cells ^23^. However, the day 4 cells could be classified as putative pre-terminal and pre-memory cells on the basis of their expression of “fate-classifier” genes associated with mature memory or TE CD8 T cells ^23^. Therefore, distinctly fated cells could be present at early times. However, it is unclear whether the distinct gene expression patterns in cells after the initial division derived from the same or different naïve parents and whether the fate-associated gene expression regimes in the single cells are reinforced in their descendants or whether they convert.

**Figure 2.**
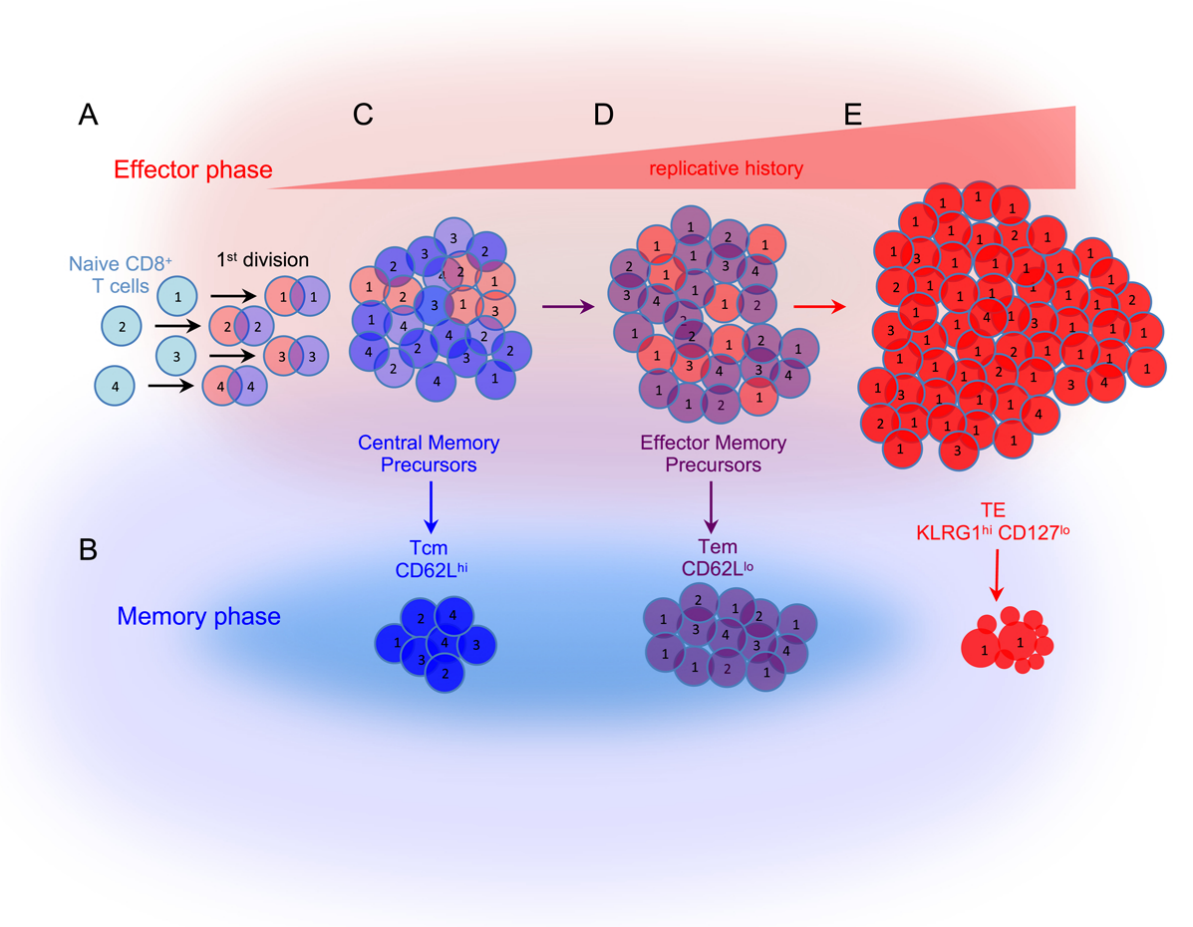
The descent of individual naïve CD8 T cells into effector and memory CD8 T-cell progeny on the basis of lineage tracing and single-cell transfer studies. ( **A**) Individual naïve CD8 T cells are recruited into the response and undergo geometric accumulation resulting in distinct CD8 T-cell families (numbers) derived from individual naïve cells. ( **B**) Each naïve cell has the potential to differentiate into progeny that exhibit central memory T (Tcm) (blue), effector memory T (Tem) (purple), or terminal effector (TE) (red) CD8 T-cell phenotypes. ( **C**) Central memory precursors (light blue) are composed of diverse families that divide slowly, ( **D**) some of which give rise to faster-dividing Tem precursors (purple). ( **E**) TE CD8 T cells comprise relatively few CD8 T-cell families that have accumulated dramatically and most die.

The ancestry of CD8 T cells at the single-cell level indicates that the overall pattern of TE and memory precursor (MP) CD8 T-cell differentiation is an average resulting from stochastic behavior of cells recruited into the response ( Figure 2). Studies applying DNA barcodes to follow CD8 T-cell families from individual naïve cells using next-generation DNA sequencing ^24^, or the transfer of individual congenically marked cells ^9, 18^, concur that the differentiated fates of single cells are highly variable ^19^. The overall response comprises relatively few clones that grow into very large CD8 T-cell families whose individual members manifest a phenotype that is indicative of shorter-lived TE CD8 T cells ( Figure 2A–E), together with many smaller CD8 T-cell families derived from a larger number of initial clones that manifest an MP CD8 T-cell phenotype that develop into most long-lived memory cells ( Figure 2B–D). These data are best fit into a model in which naïve cells differentiate linearly into MP cells that proliferate slowly and serve as precursors of more rapidly dividing Tem cells that finally give rise to shorter-lived TE CD8 T cells ^18, 19^.

## Activated naïve CD8 T cells acquire effector cell attributes before diverging into subsets with distinct potential to form memory cells

Very soon after naïve CD8 T cells become activated, they differentiate into a population of nascent CTLs that express genes which are indicative of multiple effector cell functions ^11, 25^, even though only some of these cells terminally differentiate while others give rise to memory CTLs ^14^. Moreover, although cells from early times after infection that express higher amounts of KLRG1 produce fewer memory cells, both KLRG1 ^hi^ and KLRG1 ^int^ subsets generate substantial memory cell numbers ^25^. In addition, gene expression in KLRG1 ^hi^ cells at day 5 after lymphocytic choriomeningitis virus (LCMV) infection is substantially different than in canonical TE CD8 T cells on day 8 after infection ^26, 27^, and gene expression profiles in single activated CD8 T cells 4 days after *Listeria* infection are distinct from those in single cells on day 1 after infection as well as those in single cells at the peak response on day 7 and in the memory phase ^23^. These results imply that, at early times, gene expression in the nascent CTL population is not fixed, despite having established the capacity for multiple effector functions, and that this gene program diverges as cells become TE and MP subsets as defined by KLRG1 and CD127 expression near the peak response.

The flexibility in gene expression of nascent CTLs is consistent with the stochastic nature of whether activated CD8 T cells will terminally differentiate or become memory T cells and is also born out of recent genetic experiments. An engineered reporter mouse in which Cre-recombinase is expressed from the endogenous *Klrg1* locus to activate constitutive expression of fluorescent proteins and indelibly mark cells which have expressed *Klrg1* in their history demonstrates that a substantial fraction of KLRG1 ^lo^ cells are marked with the reporter prior to the absolute peak effector response, indicating that they had previously expressed *Klrg1* and subsequently downregulated it ^28^. These “exKLRG1” cells also frequently derived from KLRG1 ^hi^ CD127 ^hi^ double-positive (DP) effector cells at the peak response and are found in all memory CD8 T-cell populations at later times ( Figure 1).

The strong memory potential of exKLRG1 cells is an indication that many, if not all, memory cells are the progeny of nascent CTLs that manifest promiscuous gene expression regimes before acquiring a more stably differentiated phenotype. This suggests that unstable gene expression in nascent CTLs facilitates differentiation along both memory and terminal differentiation paths, which are reinforced in only some progeny stochastically, a process that might be similar to multi-lineage gene expression in hematopoietic precursors which precedes and primes lineage commitment of myeloid and monocyte subsets ^29^.

## TCR stimulation rapidly induces chromatin remodeling in naïve cells which persists in differentiated effector and memory T cells

Initial TCR stimulation induces widespread alterations in chromatin accessibility of *cis*-regulatory regions prior to the initial cell division of naïve CD4 and CD8 T cells ^27, 30^. Analysis of enriched DNA motifs encoded within differentially accessible regions has provided insight into the potential transcription factors (TFs) that control the early programming of effector and memory T cells. Sequences in regions that become accessible during initial TCR stimulation in naïve cells, and that are also accessible in mature memory CD8 T cells, most frequently encode enriched motifs recognized by TFs in the RUNX, ETS, bZIP, T-BOX, IRF, RHD, PRDM1, and ZF-KLF families, which implies that these TFs might induce transcriptional reprogramming of naïve CD8 T cells, and also stabilize the differentiation of memory CD8 T cells ^27, 31– 33^. Many TFs that can bind these motifs have established functions for driving the differentiation of both effector and memory CD8 T-cell subsets and have been reviewed in detail fairly recently, but still many others have yet to be explored ^34– 36^.

The mechanism that reprograms the chromatin structure of *cis*-regulatory regions and promotes effector and memory CD4 and CD8 T-cell differentiation involves transient activation of TFs that are activated by TCR signals (that is, NFAT and AP-1), which facilitates binding of constitutively expressed or lineage-specific TFs, such as the ETS and RUNX family TFs, and presumably others ^30, 37, 38^. TCR stimulation drives transient chromatin accessibility at “inducible” regions of accessibility in conjunction with adjacent “primed” regions that remain accessible persistently after cessation of TCR signals in the differentiated progeny ^30^. Sequences within inducible regions are strongly enriched with binding sites for NFAT (RHD family) and AP-1 (bZIP family) TFs, whereas sequences within primed regions are enriched with ETS and RUNX binding sites ^30^. This process results in ETS and RUNX family TFs and presumably many others, gaining stable access to *cis*-acting regions in immune activation–relevant genes ^27, 38^.

## Chromatin remodeling of distal *cis*-regulatory regions correlates with the stability of gene expression in naïve and distinct effector and memory CD8 T-cell subsets

Analysis of chromatin accessibility in purified naïve, effector, exhausted, reinvigorated, and memory CD8 T-cell subsets indicates that an extensive accessible *cis*-regulatory landscape develops during the differentiation of both TE and MP cells, most of which is preserved in memory CD8 T cells ^31, 32, 39– 42^. Even though TE and MP CD8 T-cell populations have distinct proclivities to form memory CD8 T cells, there is considerable similarity in the chromatin accessibility profiles between both cell subsets. Consistent with the notion of a common early path of differentiation, accessibility to many of the regions from both effector cell subsets is established within the first 24 hours of TCR stimulation of naïve cells ^27, 32^. Moreover, some regions that are accessible in memory CD8 T cells but not TE cells are established by initial TCR stimulation, which indicates that specific aspects of memory CTLs are induced prior to extensive effector cell differentiation.

TE and MP CD8 T cells both manifest more accessible regions than memory CD8 T cells, when one considers regions that are also different than in naïve T cells, and most are located distal to gene transcription start sites (TSSs) ^31, 32, 42^. However, consistent with MP cells being more efficient precursors of memory CD8 T cells than TE cells, their accessibility profile is biased toward that found in memory CD8 T cells ^32^. Nevertheless, the differences in the numbers of accessible regions between MP cells and memory CTLs indicate that both chromatin condensation and chromatin opening likely occur as effector cells convert into mature memory CD8 T cells. Consistent with this, other changes to chromatin structure, such as DNA methylation, are acquired during the effector phase but are erased as MP CD8 T cells convert into memory CTLs ^15^.

The most well-defined alterations to chromatin structure that differ between effector and memory CD8 T-cell subsets appear to occur in distal intragenic regions. Distinct histone modification profiles occur at TSSs compared with transcriptional enhancers and have been used to define *cis*-regulatory function and transcriptional activity ** in *ex vivo* CD8 T-cell subsets. Chromatin immunoprecipitation and sequencing (ChIP-seq) analyses of multiple histone modifications (H3K4me3, H3K4me1, H3K27me3, and H3K27Ac) combined with algorithms trained to predict enhancer regions based on these modifications have identified many distal intergenic regions that potentially comprise enhancers in specific CD8 T-cell subsets ^42– 50^. The apparent differential activity of these putative enhancers based on histone modifications ^42, 44– 46^ and three-dimensional interactions with their target gene promoters ^44^ positively correlates with gene expression signatures of naïve, TE, and memory CD8 T cells. Thus, *cis*-regulatory regions, mainly in distal intergenic regions, undergo ** dynamic alterations as naïve CD8 T cells become activated and differentiate into distinct populations of effector and ultimately memory CD8 T cells.

Promoter proximal regulation is also likely to be important for the gene activity that defines the distinct differentiated states of CD8 T-cell subsets. Although neither differential histone modifications near TSSs ^44^ nor the accessibility of promoter-proximal regions in TE and memory CD8 T cells correlates with the differential gene expression patterns between these subsets ^32, 44^, a complete assessment of chromatin modifications that influence promoter activities has not been performed in CD8 T cells ^51^, and additional analyses could reveal important differences. In line with this idea, the occupancy of RNA polymerase II (Pol II) at the promoters of multiple effector genes differs in naïve, effector, and memory CD8 T cells ^52^, which suggests that recruitment and activity of Pol II at target gene promoters are associated with effector and memory CD8 T-cell differentiation. In addition, both subunits of P-TEFb (positive transcription elongation factor b) are essential for TE cell differentiation ^53^. P-TEFb is recruited to paused Pol II molecules at TSSs and is necessary for inducing transcriptional elongation ^54^. Therefore, overcoming Pol II pausing might be a key step that drives terminal differentiation, whereas ensuring Pol II pausing could be a mechanism that ensures that MP CD8 T cells form and perhaps the transcriptional “capacitance” of effector genes in memory CD8 T cells. Such promoter-proximal regulation is likely conferred by the differential activity and long-range interactions observed at distal *cis*-regulatory regions in distinct CD8 T-cell subsets.

## Chromatin structure and transcriptional regulation that initializes effector CTL differentiation and preserves memory CTL potential

Memory CTL differentiation involves de-activating gene expression programs of naïve cells and concurrently establishing gene expression that accounts for effector functions, tissue relocalization, persistence, and re-expansion after a secondary antigen encounter. The enrichment of Runx motifs in accessible regions that are induced during TCR stimulation and that persist in memory CTLs suggests that they might contribute to this process. Indeed, insufficiency in either *Runx3* or *Cbfb* (the partner of all three Runx TFs that is obligatory for DNA binding) impairs the acquisition of key effector functions of CTLs ^27, 55, 56^, and the activated cells do not differentiate into genuine MP CTLs or circulating memory CTLs and instead preferentially develop a TE-like phenotype ^27^. Moreover, *Runx3*-deficient cells do not repress *Tcf7* and *Bcl6*, which results in aberrant acquisition of a follicular T helper cell phenotype and trafficking into B-cell follicles ^56^. In addition, ** Runx3 deficiency impairs the differentiation of Trm cells and their homeostasis in non-lymphoid tissue (NLT) ^39^; the transcriptional control of Trm cell differentiation was recently reviewed in detail ^36^. *Runx2*-deficient T cells also exhibit defects in memory CTL generation and long-term persistence ^57^, which confirms an earlier computational prediction that Runx2 is critical for memory CD8 T-cell development ^58^. Thus, Runx family TFs drive programming of effector attributes of nascent effector CTLs and also ensure that these cells develop into memory CTLs.

Runx3 is required during TCR stimulation to establish chromatin accessibility of *cis*-regulatory regions that form stably in effector and memory CD8 T cells ^27^ and most likely depends on cooperativity with many additional TFs. The *cis*-regulatory regions that do become accessible in CD8 T cells lacking Runx3 encode many fewer motifs for RUNX, IRF, bZIP, RHD, PRDM1, and T-BOX motifs, suggesting that TFs which normally bind these sites in Runx3-sufficient cells could be collaborating factors. Runx and T-box motifs frequently co-occur within stably remodeled *cis*-regulatory regions of memory CD8 T cells, and binding regions for the two T-box TFs—T-bet and Eomesodermin—each extensively overlap those of the obligate Runx TF partner Cbfb ^33^. Together, these observations indicate that cooperativity between Runx and T-box proteins is a core regulatory mechanism that establishes the identity of effector and memory CD8 T cells ^27, 33, 55, 59^, perhaps by outcompeting nucleosomes that otherwise would form at these sites ^60^. Furthermore, the overlapping binding of Runx3, IRF4, and multiple bZIP family TFs suggests that potential cooperativity with these TFs is also important ^27^. Thus, complex cooperative interactions between multiple TFs are likely to establish a chromatin accessibility landscape during initial naïve CD8 T-cell stimulation that induces effector CD8 T-cell subsets and is stabilized in memory cells.

In addition, the *cis*-regulatory regions that are operational in Tcm cells relative to TE cells encode multiple TF motifs that predict potential TFs that promote memory CTL differentiation ^42, 44^. Binding motifs for Tcf1, Lef1, Foxo1, Foxp1, Eomes, Stat5, Gabpa, Gfi1, and Nr3c1 (as well as others) are enriched in these regions, suggesting that these TFs promote *cis*-regulatory activity that establishes and maintains memory CTL differentiation. Most of these TFs have established roles for activating gene expression that promotes T-cell quiescence, lymphoid homing, homeostasis, and the potential for self-renewal ^61– 65^. At the same time, memory CTL differentiation appears to involve actively repressing the activity of some genes to prevent terminal differentiation. RNA interference (RNAi)-mediated suppression of the glucocorticoid receptor ( *Nr3c1*) and its canonical co-repressor encoded by the chromatin regulator *Ncor1* both impaired the differentiation of MP cells and memory CTLs and increased terminal differentiation ^42^.

A somewhat paradoxical feature of memory CTL cell differentiation is that genes that promote memory CTL formation and homeostasis are initially downregulated during activation of naïve CD8 T cells, only to be re-expressed in some effector cells that become memory CTLs. The entire population of effector cells near the peak response to infection exhibits increased CpG DNA methylation genome-wide, including at representative genes such as *Il7r*, *Sell* (CD62L), and *Tcf7*, which correlates with their reduced expression in most effector CD8 T cells at the peak response ^15^. A large fraction of CD62L ^lo^ MP CD8 T cells upregulate *Sell* and undergo demethylation of its locus prior to their initial homeostatic cell division, indicating that CpG methylation is actively removed as MP cells from the effector phase convert into memory CTLs. This process does not occur at an appreciable rate in TE CD8 T cells, which remain CD62L ^lo^. CD8 T cells from mice in which the *de novo* DNA methyltransferase (Dnmt3a) was deleted early during the effector response undergo more rapid re-expression of *Il7r*, *Sell*, and *Tcf7* genes near the peak response and during the contraction phase, which indicates that initiation of DNA methylation at early times correlates with gene silencing that enforces terminal differentiation of some cells but that, in others, it can be erased at later times ^15, 66^. CD8 T cells lacking the maintenance DNA methyltransferase *Dnmt1* also appear to have reduced differentiation of effector cells, and although memory CTLs appear to form, they exhibit defective recall function ^67^. Thus, DNA methylation appears to be important for proper memory CTL differentiation, although the mechanisms that account for why some cells are able to undergo demethylation of key loci that promote memory CTL development whereas others do not and progress toward terminal differentiation are not yet clear. However, multiple chromatin reader proteins that bind methylated DNA and recruit additional chromatin-modifying factors or enzymes that chemically convert methylated cytosine residues appear to be involved. CD8 T cells from mice lacking *Mbd2*, one of four genes encoding methyl CpG-binding DNA proteins, exhibit skewing toward terminal differentiation and defective differentiation of memory CTLs that are not protective ^68^. In addition, CD8 T cells deficient in methylcytosine dioxygenase ten-eleven translocation 2 ( *Tet2*) exhibit DNA hypermethylation in multiple transcriptional regulators and enhanced memory CD8 T-cell differentiation ^69^.

## Molecular regulation that imposes terminal differentiation on effector CD8 T cells

Terminal differentiation of activated CD8 T cells positively correlates with extensive proliferative history ^19^. Even though the outcome is probabilistic at the single-cell level, the pattern of terminal differentiation of the population of cells seems to be programmed by signals received very early during activation ^18, 24^. Stimulation of T cells via their antigen receptors and co-stimulatory molecules, together with inflammatory cytokines (for example, interleukin-12 [IL-12] and IL-2), integrates to form a calculus that determines the amount of cell division in the resulting progeny ^70^. Cells accumulating larger sums of the integrated signals during priming extends their proliferative capacity and likely predisposes them to terminal differentiation ^14, 71– 74^. The same signals that induce extensive proliferation in the responding cell population also prolong their responsiveness to these stimuli, which sustains or increases the expression of TFs (such as T-bet, Zeb2, and Blimp-1) that jointly promote terminal differentiation ^14, 72, 74– 76^.

A critical feedforward transcriptional circuit involving the TFs T-bet and Zeb2 positively regulates terminal differentiation ^77, 78^. T-bet binds to the *Zeb2* locus and induces *Zeb2* expression, and both TFs appear to be necessary for optimal T-bet binding to *cis*-regulatory regions it controls; although (owing to the lack of a reliable antibody) Zeb2 occupancy was not analyzed, its putative binding motif was highly enriched within T-bet occupied regions, and T-bet binding was compromised in *Zeb2*-deficient CD8 T cells ^77^. In addition, both factors are expressed in LLE cells from the memory phase but are more highly expressed in TE cells from the peak response, which suggests that each TF has roles in both terminally differentiated and memory CTLs ^5^.

Consistent with this, memory CD8 T cells remain differentiated from TE CD8 T cells in part by preventing high expression of T-bet and Zeb2 ^14, 77^. An antagonistic relationship between the TFs Zeb1 and Zeb2 and the action of *mir-200* family microRNAs ^79^ form an important regulatory circuit that determines the memory potential of effector T cells. Zeb1 is necessary for memory CD8 T-cell differentiation and is induced in response to transforming growth factor-beta (TGF-β) signals. Together with *mir-200* family microRNAs, it represses Zeb2 expression. Runx3 also prevents high expression of T-bet and *Zeb2* that normally occurs in TE cells ^27^.

In addition, the bZIP family TF, Bach2, deactivates terminal CTL differentiation by preventing TCR-induced AP-1–driven signals by competing with Jun proteins for DNA occupancy within *cis*-regulatory regions ^58, 80^ and is necessary for exKLRG1 cells to develop into memory CTLs ^28^. Runx3 appears to contribute to this process because *Runx3*-deficient CD8 T cells fail to induce chromatin accessibility of *cis*-regulatory regions encoding ** Bach2-binding motifs ^27^. Therefore, negative feedback is provided by TFs that initially drive acquisition of effector cell attributes during CD8 T-cell activation which prevents terminal differentiation.

Terminal CTL differentiation involves stable repression of genes encoding stem cell–like qualities that normally promote the long-lived nature of Tcm cells ^64, 81, 82^. Both T-bet and Zeb2 repress features of memory CD8 T cells (for example, by binding directly to the *Il2* and *Il7r* genes and repressing their expression). In addition, high Blimp-1 expression causes repression of Id3, which retards the ability of effector cells to contribute to the memory CTL compartment ^83, 84^. Also, chromatin-level repression of genes that promote lymphoid homing and quiescence and other features of “stemness” that can be considered “pro-memory” promotes commitment to terminal differentiation ^15, 26, 48, 85^. Methylation of histone H3K9 and H3K27 is a well-studied mechanism that promotes chromatin condensation and gene silencing during cell development ^86^. Upon activation, naïve CD8 T cells rapidly accumulate islands of histone H3K9 trimethylation (H3K9me3), especially at genes such as *Il7r* and *Sell* ^85^. H3K9me3 is deposited by multiple methyltransferases, including the suppressor of variegation 3-9 homolog 1 ( *Suv39h1*), and is a histone modification that recruits multiple proteins in the chromobox (Cbx) family to bind adjacent nucleosomes together, a process that reinforces recruitment of additional Suv39h1 and promotes spreading of H3K9me3 deposition ^87, 88^. In addition, Suv39h1 interacts with Mbd family proteins, which suggests that DNA methylation could instigate or enhance Suv39h1 recruitment and H3K9me3 deposition ^89^. *Suv39h1*-deficient CD8 T cells fail to repress naïve and stem cell–associated genes and exhibit a loss in the inverse correlation between H3K9me3 density and stem cell gene expression ^85^. These cells accumulate poorly and develop a normal TE CD8 T-cell phenotype inefficiently, and the resulting memory cells are not protective ^85^.

Similarly, repression of MP cell signature genes by enhanced deposition of H3K27me3 in *cis*-regulatory regions of TE CD8 T cells also promotes terminal differentiation ^23, 48^. H3K27me3 deposition is catalyzed by the methyltransferase Ezh2, which is upregulated upon stimulation of naïve CD8 T cells ^48^, and its mRNA is more highly expressed in a subset of responding CD8 T cells classified as pre-terminal effector cells ^23^. Disruption of *Ezh2* impairs CD8 T-cell accumulation and effector cell differentiation ^23, 48^. This phenotype correlates with reduced H3K27me3 and enhanced expression of *Eomes*, *Tcf7*, and *Klf2* genes, which encode TFs that promote competitive fitness of Tcm cells, their maintenance, and lymphoid retention ^23, 61, 65, 90^. Thus, coordinated targeting of histone methyltransferase activity in effector cells leads to methylation of H3K9 and H3K27 residues in nucleosomes of genes that are essential for memory cell homeostasis, which represses their expression and may ensure terminal differentiation.

Finally, the stability of phenotypes in CTL subsets, as in many other developmental systems, is enforced by TFs that drive particular cell states by continuously directing the activity of chromatin regulators to their appropriate gene targets ^91^. In the earliest part of the memory phase, LLEs that are KLRG1 ^hi^ retain properties that endow them with additional effector capacities and persistence at early times during the memory phase ^5^ ( Figure 1). The phenotype of these cells depends on continued expression of the proteins Id2 and Zeb2 ^6, 78^. Conditional disruption of *Id2* in KLRG1 ^hi^ cells after differentiation of LLE results in loss of KLRG1 expression and in conversion of their transcriptional profile into one reminiscent of that found in Tcm cells ^6^. These results demonstrate that the persistent activity of certain TFs is essential for maintaining the differentiated state of memory CTLs after they have been generated. Thus, while these differentiation programs depend on chromatin remodeling, they are maintained by the continuous activity of specific TFs.

## Toward a unified model of memory CD8 T-cell differentiation

**Figure 3.**
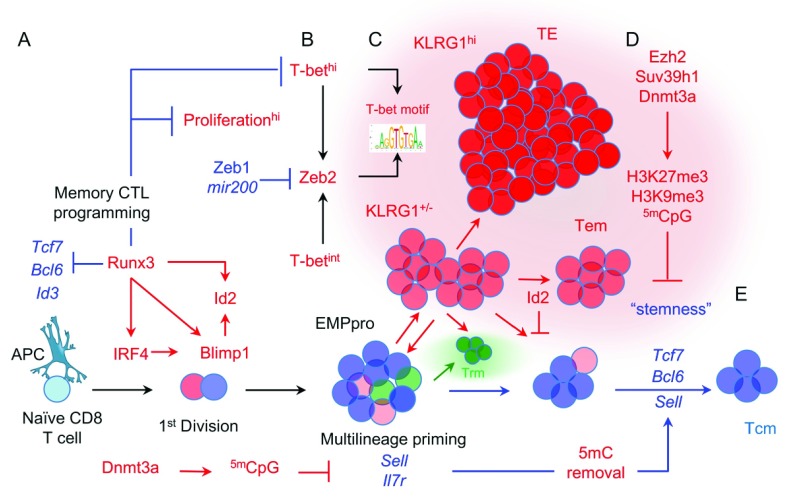
An integrated model of memory CD8 T-cell differentiation. ( **A**) Individual naïve CD8 T cells undergo memory CD8 T-cell programming wherein they acquire fundamental traits of fully developed memory CD8 T cells prior to the first cell division. ( **B**) The growing nascent CD8 T-cell population comprises a transitional population of effector and memory precursor progenitor (EMPpro) cells that are metastable at the chromatin and transcriptional level and can give rise to all subsets of differentiated CD8 T-cell progeny. ( **C**) A small number of EMPpro cells randomly undergo massive proliferation coupled to higher expression of multiple transcription factors (TFs) in response to inflammatory signals that drive transcription underlying the phenotypic and functional profiles of terminally differentiated CD8 T cells. ( **D**) Multiple chromatin regulatory factors that methylate DNA and histones persistently repress genes that otherwise favor quiescence, lymphoid retention, and overall “stemness” and thus enforce terminal differentiation. Factors generally associated with terminal CD8 T-cell differentiation (red) and memory (blue) are highlighted by color but are not intended to imply exclusive correlations.

Several models have been proposed to conceptualize how naïve CD8 T cells differentiate into memory CD8 T cells ^19, 35, 92^. An amenable solution that bears similarity to the decreasing potential and progressive differentiation models but that includes insight from single-cell tracing studies and population analyses of chromatin structure suggests that naïve CD8 T cells rapidly acquire critical features of both effector and memory CD8 T cells upon TCR activation and thus comprise effector and memory precursor progenitor (EMPpro) cells ( Figure 3). Cells in the EMPpro population manifest metastable transcriptional states characterized by promiscuous gene expression among individual cells and stochastic proclivity for acceleration or diversion into effector memory–like cells and further commitment to extensive proliferation and terminal differentiation, or reversion to a slowly dividing EMPpro state, relaxation into MP cells, and ultimately differentiation into memory CD8 T cells. Between these extremes, some cells depart the spleen and seed peripheral NLTs to form precursors of Trm CD8 T cells ^39^. The probability that cells opt to proliferate extensively and differentiate into TE CTLs is influenced by the integration of signals that individual naïve cells experience during initial activation. In addition, signals in the local microenvironment as the nascent EMPpro families accumulate may sustain or antagonize these signals in some cells ^93^, and influence the binding activity of specific TFs and alterations to chromatin structure that drive the gene expression programs specific to TE and MP cells, thus progressively reinforcing (or reversing) the fates of individual cells that tend to diverge along these pathways to memory. Therefore, even though individual T cells arrive at their fates randomly, the patterns of memory CTL differentiation are influenced deterministically.

## Abbreviations

CTL, cytotoxic T lymphocyte; EMPpro, progenitor effector and memory precursor; H3K9me3, histone H3 lysine 9 tri-methylation; H3K27me3, histone H3 lysine 27 tri-methylation; IL, interleukin; LLE, long-lived effector; MP, memory precursor; NLT, non-lymphoid tissue; Pol II, polymerase II; P-TEFb, positive transcription elongation factor b; SLO, secondary lymphoid organ; Tcm, central memory; TCR, T-cell receptor; TE, terminal effector; Tem, effector memory; TF, transcription factor; Trm, tissue-resident memory; TSS, transcription start site.
